# SOS2 Comes to the Fore: Differential Functionalities in Physiology and Pathology

**DOI:** 10.3390/ijms22126613

**Published:** 2021-06-21

**Authors:** Fernando C. Baltanás, Rósula García-Navas, Eugenio Santos

**Affiliations:** Lab. 1, CIC-IBMCC, Universidad de Salamanca-CSIC and CIBERONC, 37007 Salamanca, Spain; rosula@usal.es (R.G.-N.); esantos@usal.es (E.S.)

**Keywords:** son of sevenless, SOS1, SOS2, RAS signaling, GEFs

## Abstract

The SOS family of Ras-GEFs encompasses two highly homologous and widely expressed members, SOS1 and SOS2. Despite their similar structures and expression patterns, early studies of constitutive KO mice showing that SOS1-KO mutants were embryonic lethal while SOS2-KO mice were viable led to initially viewing SOS1 as the main Ras-GEF linking external stimuli to downstream RAS signaling, while obviating the functional significance of SOS2. Subsequently, different genetic and/or pharmacological ablation tools defined more precisely the functional specificity/redundancy of the SOS1/2 GEFs. Interestingly, the defective phenotypes observed in concomitantly ablated SOS1/2-DKO contexts are frequently much stronger than in single SOS1-KO scenarios and undetectable in single SOS2-KO cells, demonstrating functional redundancy between them and suggesting an ancillary role of SOS2 in the absence of SOS1. Preferential SOS1 role was also demonstrated in different RASopathies and tumors. Conversely, specific SOS2 functions, including a critical role in regulation of the RAS–PI3K/AKT signaling axis in keratinocytes and KRAS-driven tumor lines or in control of epidermal stem cell homeostasis, were also reported. Specific SOS2 mutations were also identified in some RASopathies and cancer forms. The relevance/specificity of the newly uncovered functional roles suggests that SOS2 should join SOS1 for consideration as a relevant biomarker/therapy target.

## 1. SOS2 vs. SOS1 Function: An Introductory Timeline Perspective

### 1.1. Ras GEFs and the SOS Family

**Figure 1:**
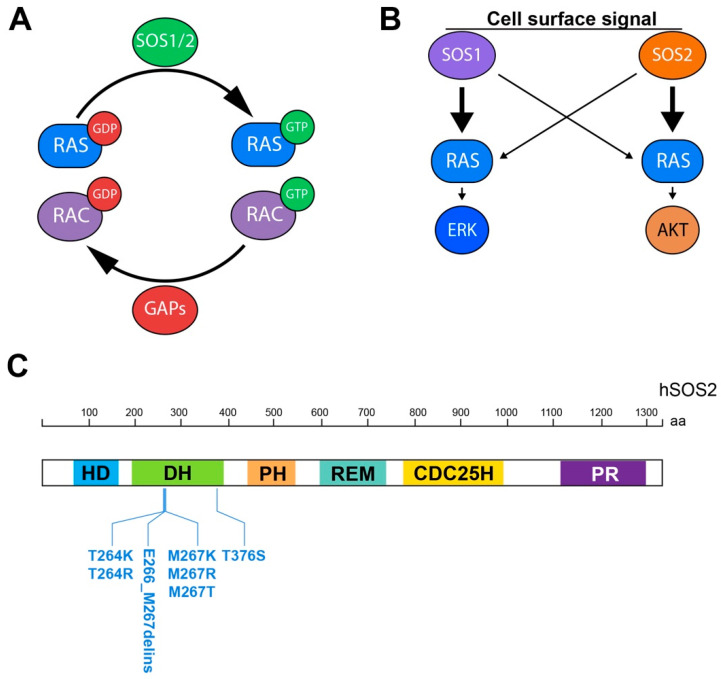
**Functional relevance of SOS1/2 GEFs in physiological RAS signaling pathways and RASopathies.** (**A**) Schematic representation of RAS and RAC activation/deactivation cycle mediated by SOS1/2 GEFs and GAPs, respectively. (**B**) Differential participation of SOS1 and SOS2 in downstream RAs signaling as suggested by current experimental evidence. (**C**) SOS2-specific mutational landscape in human RASopathies (Noonan syndrome, NS9 type). HD: histone domain; DH: Dbl homology; PH: pleckstrin homology; CDC25H: cell division cycle 25 homology; REM: RAS exchange motif; PR: proline-rich.

The proteins of the RAS superfamily are small GTPases known to shift between inactive (GDP-bound) and active (GTP-bound) conformations in a cycle regulated by activating Guanine nucleotide Exchange Factors (GEFs) that facilitate GDP/GTP exchange, and deactivating GTPase activating proteins (GAPs) that multiply their intrinsic GTPase activity (Figure 1A) [1,2,3,4].

Three main Ras-GEF families (RasGRF 1/2, SOS 1/2, and RasGRP l–4) have been described in mammalian cells with the ability to promote GDP/GTP exchange on the members of the RAS subfamily, and also some members of the RAC subfamily of small GTPases [5,6,7,8]. All mammalian Ras-GEFs share the presence of catalytic CDC25H and REM modules in their primary sequences but, otherwise, each GEF family displays markedly distinct patterns of protein structure, function, regulation, and tissue expression. The members of the GRF family act preferentially, but not exclusively, in cells of the central nervous system [6,9,10], whereas the GRP family members function mostly in hematological cells and tissues [11,12]. In contrast, the members of the SOS (Son of sevenless) family are the most universal Ras-GEF activators, being recognized as the most widely expressed and functionally relevant GEFs with regards to RAS activation by various upstream signals in mammalian cells [5]. The SOS family encompasses two highly homologous, ubiquitously expressed members (SOS1 and SOS2) functioning in multiple signaling pathways involving RAS or RAC activation downstream of a wide variety of cell surface receptors [5,13].

The initial characterization of the first available constitutive knockout (KO) mouse strains of the SOS family showed that SOS1 ablation causes mid-embryonic lethality in mice [14,15], whereas constitutive SOS2-KO mice are perfectly viable and fertile [16]. Because of this and the stronger phenotypic traits associated to SOS1 ablation, most early functional studies of the SOS family focused almost exclusively on SOS1, and rather little attention was paid to analyzing the functional relevance of SOS2 [5]. The view that SOS1, but not SOS2, is the key GEF family member in RAS-signal transduction in metazoan cells was also probably behind the long search for, and development of, specific, small-molecule SOS1 inhibitors that have recently reached preclinical and clinical testing against RAS-driven tumors [5,17,18].

### 1.2. Functional Redundancy/Specificity of SOS2 vs. SOS1

Despite the earlier lack of focus on the functional relevance of SOS2, many subsequent studies have uncovered specific functions unambiguously attributed to SOS2 in different physiological and pathological contexts that clearly document the functional specificity of this particular SOS GEF family member.

In particular, the development, about 8 years ago, of conditional, tamoxifen-inducible, SOS1-null mutant mice made it possible to bypass the embryonal lethality of SOS1-null mutants and opened the way to carry out relevant functional studies of SOS2 by allowing biological samples originated from adult mouse littermates of four relevant SOS genotypes (WT, SOS1-KO, SOS2-KO and SOS1/2-DKO) to be generated and functionally compared [19]. Somewhat surprisingly, adult SOS1-KO or SOS2-KO mice were perfectly viable, but double SOS1/2-DKO animals died very rapidly [19], demonstrating a critical contribution of the SOS2 isoform (at least when SOS1 is absent) at the level of full organismal survival and homeostasis, and thus opening new avenues for consideration of SOS2 as a functionally relevant player in mammalian RAS signaling pathways. In this regard, a number of recent functional studies of SOS1 and SOS2 using diverse genetic and pharmacological SOS ablation approaches have significantly clarified, during the last decade, the mechanistic details underlying the functional specificity/redundancy of the SOS1 and SOS2 GEFs in a wide array of tissues and cells, both under physiological and pathological conditions [20,21,22,23,24,25] (see [5] for a review).

Specifically, detailed functional comparisons between primary mouse embryonic fibroblasts (MEFs) extracted from SOS1-KO and/or SOS2-KO mice have documented a dominant role of SOS1 over SOS2 regarding the control of a series of critical cellular physiological processes, including cellular proliferation and migration [20,21], inflammation [22], and maintenance of intracellular redox homeostasis [20,26]. The functional prevalence of SOS1 is not limited to the above-mentioned physiological contexts, but has also been demonstrated under different specific pathological contexts. In particular, a specific, critical requirement of SOS1 was demonstrated for development of BCR–ABL-driven leukemia [24,27], as well as in skin homeostasis and chemically induced carcinogenesis [21,28]. Likewise, both SH2P and SOS1 have been shown to be essential signaling mediators in wild-type KRAS-amplified gastroesophageal cancer [5,29].

### 1.3. Hierarchy of Action of the SOS Family Members

As described above, most reports support the functional dominance of SOS1 over SOS2 regarding their participation in control of several major intracellular processes, such as proliferation, migration, inflammation, or regulation of intracellular ROS levels [20,25]. Remarkably, in all those processes, the defective cellular phenotypes observed in SOS1/2-DKO samples are always much stronger than in single SOS1-KO cells, while undetectable in single SOS2-KO contexts, suggesting a specific, ancillary role of SOS2 that only becomes easily visible in the absence of SOS1 [19,20,21,30].

Regarding the participation of SOS1 and SOS2 in Ras signaling pathways, the initial analyses of constitutive SOS1-KO mouse embryo fibroblast (MEF) cell lines indicated that SOS1 (but not SOS2) is required for long-term activation of the Ras-ERK pathway, with SOS1 participating in both short-term and long-term signaling, while SOS2-dependent signals are predominantly short-term [14]. More recent studies analyzing inducible SOS1-KO biological samples in mouse keratinocytes also support that view [25] and have also confirmed that SOS1 is the dominant player regarding the process and kinetics of RAS activation (GTP loading) upon cell stimulation by various upstream signals and growth factors [20,25]. Of relevance also are other recent studies in cell lines devoid of SOS1 and/or SOS2 that have described the specific, primary involvement of SOS2 in regulation of the PI3K/AKT signaling axis, whereas SOS1 appears to be the dominant player in the MEK/ERK signaling axis [25,30,31,32]. Furthermore, regarding SOS2 functional specificities in cellular pathological contexts, a hierarchical requirement for SOS2 to mediate RAS-driven cell transformation has also been reported recently in certain cell populations [31,32].

### 1.4. Distinct Functional Roles of SOS2 and SOS1 in the Skin and Epidermal Cancers

EGF-dependent RAS–RAF signaling has been shown to be essential for epidermal development and carcinogenesis [33,34,35]. In this regard, it was also shown that SOS1 upregulation resulted in development of skin papillomas with 100% penetrance, supporting a critical role of SOS in this process in epidermal cells [28].

More recently, our laboratory has also characterized/analyzed in detail the specific involvement of SOS1 and/or SOS2 in homeostasis of the skin, as well as in tumoral and nontumoral skin pathologies [21,25]. Our initial studies in adult KO animal models showed that SOS1 ablation (but not SOS2 ablation) produced significant alterations of the overall layered structure of the skin in adult mice, although, interestingly, these skin architectural defects were markedly worsened when both SOS1 and SOS2 proteins were concomitantly ablated [21]. Furthermore, the skin of adult SOS1-ablated mice and, more markedly, SOS1/2-DKO mice showed a severe impairment of its physiological ability to repair skin wounds, as well as almost complete disappearance of the neutrophil-mediated inflammatory response in the injury site. In addition, SOS1 disruption (but not SOS2 ablation) delayed the onset of tumor initiation, decreased tumor growth, and prevented malignant progression of papillomas when using the known DMBA/TPA model of chemically induced skin carcinogenesis in mice [21].

While these observations demonstrated that SOS1 is clearly predominant with regards to skin homeostasis, wound healing, and chemically induced skin carcinogenesis, it still remained unclear whether the defective phenotypes observed in the skin of SOS1-deficient mice were cell-autonomous or depended on their local manifestation in specific cell compartments of the skin. We have addressed these questions in a recent report involving extensive detailed analyses of the specific subpopulation of keratinocytes present in the skin of both newborn and adult SOS1-KO and/or SOS2-KO mice [25]. While these studies confirmed the prevalent role of SOS1 over SOS2 in regulation of the proliferation of primary mouse keratinocytes, our detailed analyses of primary keratinocytes derived from newborn and adult mice of four relevant SOS genotypes (WT, SOS1-KO, SOS-KO, and SOS1/2-DKO) uncovered previously unrecognized functional contributions of SOS2 regarding skin architecture, as well as proliferation, differentiation, and survival of primary keratinocytes [25]. In particular, our analyses uncovered a specific, significant reduction of the stem cell population located in skin hair follicles of both newborn and adult SOS2-KO mice [21]. As this population is essential for replacing, restoring, and regenerating the mouse epidermis, these data confirm that SOS2 plays specific, cell-autonomous functions (distinct from those of SOS1) in keratinocytes, and reveal a novel, essential role of SOS2 in control of epidermal stem cell homeostasis [21,25].

### 1.5. Differential Involvement of SOS2 and SOS1 in Cellular Pathological Contexts

Growing experimental evidence has accumulated in recent years that supports the functional implication of SOS GEFs in human tumors and other RAS-related pathologies. So far, a predominant occurrence of SOS1 genetic alterations has been reported in most pathological contexts involving SOS GEFs. In this regard, a significant number of gain-of-function SOS1 mutations (and, more rarely, SOS2 mutations), resulting in subsequent hyperactivation of RAS signaling, have been identified in inherited RASopathies, such as Noonan syndrome (NS) or hereditary gingival fibromatosis, as well as in various sporadic human cancers, including endometrial tumors and lung adenocarcinoma, among others [5]. However, during the last few years, a previously undetected but relevant involvement of SOS2 in some of these pathologies is also coming to light in a series of studies describing specific SOS2 gene alterations that have been identified in several forms of cancers and RASopathies, as well as the potential therapeutic effect of explicit SOS2 removal in certain tumor cell lines [36,37,38,39,40]. All in all, these observations and the above-described timeline of experimental evidence support the notion that, besides SOS1, SOS2 may also constitute a worthy therapy target for prevention and/or treatment of some specific tumor and nontumor pathologies with epidermal origin or dysregulated PI3K/AKT signal transmission [25].

### 1.6. SOS1/2 Inhibitors in Pathological Settings

RAS oncoproteins were sometimes considered “undruggable” in the past, but that notion has been proven wrong by the development of promising inhibitors that are currently being characterized at different stages of preclinical and clinical testing [5,41]. In addition, a renewed interest has recently emerged to target SOS proteins in an effort to attenuate oncogenic signaling in tumors harboring altered RTK–RAS–ERK signaling pathways (Table 1). In this regard, new small-molecule SOS1 inhibitors have been obtained in the last few years with the ability to either (i) interfere with the functional SOS:RAS interactions, or (ii) to limit the intrinsic GEF activity of SOS1 protein [5] (Table 1).

Regarding the first group, one of the most promising direct RAS inhibitors developed so far is the KRAS^G12C^ inhibitor AMG510, recently named Sotorasib in clinical settings [42]. In particular, a phase 1 trial (NCT04185883; https://clinicaltrials.gov/ct2/show/NCT04185883 (accessed on 20 June 2021)) described Sotorasib anticancer activity in patients with KRAS^G12C^-mutated advanced solid tumors, with a particularly potent beneficial effect in patients with non-small-cell lung cancer (NSCLC) [43]. In addition, a phase 2 clinical trial (NCT03600883; https://clinicaltrials.gov/ct2/show/NCT03600883 (accessed on 20 June 2021); Table 1), showed that Sotorasib therapy resulted in a long-term clinical benefit in patients with previously treated KRAS^G12C^-mutated NSCLC [44]. Finally, a randomized phase III trial (NCT04303780; https://clinicaltrials.gov/ct2/show/NCT04303780 (accessed on 20 June 2021)) currently recruiting patients is devoted to comparing Sotorasib with docetaxel in advanced NSCLC patients with KRAS^G12C^ mutation who have progressed after combination of platinum-based chemotherapy and checkpoint inhibitor [45].

Regarding the group of small-molecule, direct SOS inhibitors, only drugs designed to act against SOS1 are available at this moment, whereas inhibitors specifically acting on SOS2 are not yet described [5,46] (Table 1). Within this group, BAY-293 has been shown to bind directly to the SOS partner of the RAS:SOS complex, thus preventing KRAS–SOS1 complex formation [47]. Recent reports have described the therapeutic effect of BAY-293 in EGFR-mutated tumor cell lines, and also its synergistic action with Osimertinib [30] and KRAS^G12C^ inhibitors [47]. A weakness of this compound is that its effect has been proven in vitro but not in vivo [47]. On the other hand, BI-3406, the first-in-class, orally bioavailable, in vivo tested, direct SOS1-inhibitor elicits activity against many KRAS variants, including all major G12 and G13 oncoproteins, and demonstrates synergistic therapeutical effects if combined with MEK inhibitor [23]. Moreover, a combination of BI-3406 and trametinib has potent activity against secondarily acquired resistance due to new KRAS mutations [48]. Finally, a phase I clinical trial (NCT04111458; https://clinicaltrials.gov/ct2/show/NCT04111458 (accessed on 20 June 2021); Table 1) has also recently been started with BI-1701963 (a compound which exhibits high similarities in its mode of action with BI-3406) [46] that is focused on patients with advanced KRAS-mutated cancers, in order to evaluate safety, tolerability, pharmacokinetics, and pharmacodynamic properties (Table 1). It will be interesting to determine in the future whether SOS1 inhibitors can also block SOS2 function, and vice versa.

**Table 1:** **Inhibitors of SOS GEF function in pathological contexts.** List of compounds and experimental evidence documenting their ability to disrupt functional interactions of SOS and RAS targets in RAS:SOS complexes, or to directly inhibit the intrinsic GEF activity of SOS proteins.

Compound	Mode of Action	Preclinical/Clinical Trial Identifier	Reference
Sotorasib (AMG510)	KRAS^G12C^ inhibitor	NCT04185883 NCT03600883 NCT04303780	[43,44,45]
BAY-293	SOS1 inhibitor	Preclinical	[30,47]
BI-3406	SOS1 inhibitor	Preclinical	[23]
BI-1701963	SOS1 inhibitor	NCT04111458	https://clinicaltrials.gov/ct2/show/NCT04111458 (accessed on 20 June 2021)

The following sections in this review focus on different aspects of SOS2 function in various physiological processes and pathological contexts, and also pinpoint some remaining questions still requiring further clarification about potential, specific functional role(s) of SOS2. It is apparent that further, comprehensive functional analysis of specific tissue/cell lineages will be needed to fully unveil the specific functional contributions of SOS2 in various health and disease contexts. Although SOS2 was frequently considered in the past as the “ugly duckling” of the SOS family, the more recent and complete studies of the regulatory and functional aspects of the SOS family members support the notion that SOS2 may well become a “swan”.

## 2. SOS2 and SOS1: So Similar but So Functionally Different. Some Mechanistic Considerations

As mentioned above, despite their remarkable homology, it is apparent that SOS1 is critically required for more functionally relevant roles than SOS2, but very little is known about the precise mechanistic reasons explaining the noticeable functional differences observed between both SOS isoforms in different physiological cellular contexts.

An initial, simplistic consideration in the search for mechanistic explanations might dwell on the analysis of potential differences of expression levels between SOS1 and SOS2 in different biological contexts. For example, the initial detection of high expression levels of SOS1 mRNA and protein in placental labyrinth trophoblasts, whereas SOS2 levels were significantly lower [14], offered a likely explanation for the observation that SOS2 presence is not sufficient to rescue the mid-gestation lethality caused by the absence of SOS1 in constitutive SOS1-KO mice [14]. In contrast, the fact that SOS1 and SOS2 are almost ubiquitously expressed at significant intracellular concentrations in most postembryonal organs/tissues/cells examined [5] indicates that mechanisms other than expression level may account for the dominant role of SOS1 regarding cellular proliferation, migration, inflammation, or control of intracellular redox homeostasis [19,20,21,26,30]. Interestingly, despite the seemingly prevalent functional contributions of SOS1 in comparison to SOS2, analysis of large database sets of available microarray hybridization expression data shows the presence of higher amounts of SOS2 transcripts than of SOS1 transcripts in different cellular settings [21,25]. In any case, it is apparent that a definitive quantitation of the steady-state, real intracellular concentration of SOS1 and SOS2 in different biological contexts can be achieved only by accurate mass-spectrometric determination and quantitation of the amounts of specific peptides unique for either SOS1 or SOS2 in each sample analyzed. In this regard, a recent proteomic study performed across different cell types has revealed that the absolute abundance of SOS1 and SOS2 proteins is quite similar [49].

Another relevant consideration regarding the mechanistic basis of the functional specificities shown by the SOS1 and SOS2 Ras-GEFs is the existence of distinct, specific transcriptional programs specifically linked to the expression in cells of each one of these two otherwise highly homologous family members. Curiously, most SOS-related transcriptional data accessible in public databases deal with SOS1-dependent transcriptomic alterations networks observed in various native or drug-treated tumors and pathologies [5,23,50,51], and much less information is available regarding the characterization of the specific transcriptional networks driven by the presence of SOS1 or SOS2 in different cellular physiological contexts (SOS1: https://www.ncbi.nlm.nih.gov/gds/?term=sos1 (accessed on 20 June 2021); SOS2: https://www.ncbi.nlm.nih.gov/gds/?term=sos2 (accessed on 20 June 2021)). In this regard, our comparison of transcriptional networks of primary cells derived from SOS1-KO and/or SOS2-KO mice has revealed a remarkably higher impact of SOS1 ablation than SOS2 ablation on the resulting transcriptomic profiles. Interestingly, we observed that SOS2 depletion resulted in practically negligible alterations as compared to SOS1 ablation in primary MEFs (unpublished) and keratinocytes [25]. Furthermore, as with other phenotypic alterations [19,20,21,22,23,25], concomitant ablation of SOS1 and SOS2 caused significantly higher alterations of the transcriptional patterns than single SOS1 depletion, suggesting a possible adjuvant role of SOS2 in this regard when SOS1 is already absent. These observations underscore a significantly prevalent role of SOS1 over SOS2 regarding the transcriptional regulation of cellular proliferation and differentiation processes, at least in primary cells of mice [25].

A number of biochemical differences between SOS1 and SOS2 GEF proteins that have been reported in the literature [5] are also likely to be highly significant factors contributing to the different functionalities exhibited by these two isoforms in different biological contexts. Among other functional aspects, these different biochemical properties are thought to impact on the protein half-life and the intracellular stability and homeostasis of the SOS1 and SOS2 GEF proteins, as well as on the various protein–protein interactions (PPI) in which they can engage under different biological conditions. For example, it has been reported that hSOS2 has a higher affinity for the adaptor protein GRB2 than hSOS1 [52], or that SOS1 proteins are more stable than SOS2 proteins since the latter seem to be degraded by a ubiquitin- and 26S proteasome-dependent process in mouse cells [53,54]. Separate studies have also shown that SOS2 binds less efficiently than SOS1 to EGFR and Shc in EGF-treated cells, and that SOS2-dependent signals are predominantly short-term, whereas SOS1 participates in short- and long-term signaling upon receptor stimulation [14,25]. Furthermore, a recent report has also described specific in vivo direct interactions of SOS1 with the CSN3 subunit of the COP signalosome and PKD, which may contribute to homeostatic control of intracellular RAS activation [55]. In this regard, it will be of interest to determine in future whether or not SOS2 may also bind to CSN3.

Differences in 3D structure and regulation may also contribute to the differential functionality of SOS1 and SOS2. The allosteric binding of RAS•GTP to the SOS1 REM domain was clearly shown to relieve SOS1 autoinhibition and create a positive feedback loop of RAS activation, thus altogether increasing the catalytic activity of SOS1 [5,56]. However, the scope and significance of the potential allosteric activation of SOS2 via its own REM domain remains undefined at this time [57]. More extensive analyses of full-length SOS2 protein crystals are bound to provide additional valuable information in this regard in the future.

### 2.1. Is SOS2 a Bona Fide Rac-GEF In Vivo?

Many prior reports have documented the ability of the SOS GEFs to act as bifunctional GEF activators capable of activating not only all members of the RAS protein family, but also some members of the RAC family of proteins. In view of this, some functional disparities displayed by SOS1 and SOS2 in different cellular contexts might also be linked, at least in part, to their specific, potentially differential participation in processes of activation of RAS and/or RAC intracellular proteins upon cellular stimulation by different external signals [5,13,20,24].

Mechanistically, the SOS GEFs are known to promote signal internalization and subsequent RAS/RAC activation through a process involving their recruitment from the cytosol to the plasma membrane via complex formation with different adaptor proteins (refs). In the context of this mechanistic model, the differential activity of SOS over RAS or RAC targets in vivo appears to be mediated by mutually exclusive interactions with either GRB2 or E3B1, respectively [5]. Although the precise mechanistic details remain yet poorly understood, it is generally accepted that SOS-mediated activation of RAC requires recruitment of SOS–E3B1 complexes to actin filaments found within membrane ruffles, thus facilitating RAC activation by the DH (Dbl homology) domain. So far, only SOS1 has been formally demonstrated to act as a bona fide Rac-GEF [13,58], and the hypothetical function of SOS2 as an Rac-GEF awaits future, stronger experimental evidence. In any case, the high homology shared by SOS1 and SOS2 in their overall modular protein structure/sequence and, in particular, in their DH domains responsible for RAC activation (overall 84% similarity and 70.6% amino acid identity) [5], together with the experimental demonstration of physical interaction between hSOS2 and hE3B1 in COS cells [59], support the notion of postulating SOS2 as a potential RAC activator, at least in certain cellular contexts. Interestingly, direct analysis of primary SOS1/2 KO primary MEFs has shown that single ablation of either SOS1 or SOS2 did not impair the overall level of EGF-dependent RAC activation, whereas combined SOS1/2 depletion significantly reduced the levels of RAC activation [20], suggesting functionally redundant contributions of SOS1 and SOS2 with regards to RAC activation after EGF stimulation [20]. Additional mechanistic studies are needed to fully ascertain the potential role of SOS2 as an RAC-GEF activator in a variety of cellular contexts.

### 2.2. SOS2 as a Key Modulator of PI3K–AKT Signaling

After surface receptor stimulation and subsequent SOS-mediated RAS activation, the GTP-loaded RAS proteins are known to activate various downstream signaling pathways which are essential for the control of a wide variety of cellular processes. The RAF1/mitogen-activated protein kinase (MAPK)/extracellular signal-regulated protein kinase (ERK) signaling pathway is crucial for the control of many cellular events, including proliferation, transformation, or survival. Furthermore, downstream RAS signaling through phosphoinositide 3-kinase (PI3K) has also been shown to have an essential role in processes such as cell survival, cytoskeleton reorganization, cell motility or invasiveness, among others [60]. Since a well-regulated balance between the RAS–ERK and RAS–PI3K signaling axes is essential for adequate cellular signaling homeostasis, it will be relevant in this regard to elucidate the relative functional contributions of SOS1 and SOS2 to either signaling axis in different cellular contexts [5]. Interestingly, our functional analyses of RAS activation and downstream signaling in primary keratinocytes from WT and SOS1/2-ablated genotypes has recently revealed a prevalent role of SOS1 regarding control of RAS activation (GTP loading), and a mechanistic overlapping of SOS1 and SOS2 regarding cell proliferation and survival in response to EGF, with dominant contribution of SOS1 to the RAS–ERK axis and SOS2 to the RAS–PI3K/AKT axis [25]. These recent observations in keratinocytes confirm and extend previous reports in primary MEFs and in a wide array of tumor cell lines that also demonstrated a preferential role of SOS1 in the control of cell proliferation and activation of the RAS–ERK pathway [5,20,21].

The notion of specific, relevant functional cellular roles played by SOS2 is firmly supported by studies from R. Kortum´s lab demonstrating that SOS2 promotes EGF-stimulated AKT phosphorylation in cells expressing mutant RAS. In particular, single SOS2 ablation or silencing in a variety of mouse and human cell lines results in significant reduction of AKT, but not ERK, phosphorylation and ability for anchorage-independent growth in RAS-mutant cells [31,32] (Table 2). The same lab has also reported the potential involvement of SOS2 in SHP2-mediated signaling pathways [30]. Overall, the observation that SOS2-dependent PI3K/AKT signaling appears to be crucial for transformation in cells harboring mutant KRAS genes [31,32] suggests that SOS2 could be considered as a potential therapy target in KRAS-driven oncogenic processes with dysregulated PI3K/AKT signal transmission (Figure 1B).

## 3. SOS2 Functional Role(s) in Pathological Contexts

The specific, functional involvement of SOS2 in different pathologies has also been recently reported, although with lower incidence rates than for SOS1 [5]. Pathologies linked to SOS2 alterations include different inherited proliferative/developmental disorders (RASopathies), as well as sporadic tumors and other nontumoral diseases.

### 3.1. SOS2 in Noonan Syndrome

The RASopathies comprise a defined group of inherited developmental syndromes with partially overlapping clinical features linked to germline mutations affecting different members of the RAS–ERK pathway [61]. The most common RASopathy, Noonan Syndrome (NS), is an autosomal dominant condition whose features may include distinctive facial appearance, short stature, broad or webbed neck, congenital heart defects, bleeding problems, skeletal malformations, as well as physical and neurodevelopmental delays and cognitive deficits [62].

SOS1 is the second most frequently mutated gene in this syndrome (~16.5% of cases; up to 70 different mutations described [5,63]). Whereas early screenings reported only SOS1 mutations [62,63], more recent studies have identified a number of SOS2 mutations, including missense activating mutations in seven specific residues located in the SOS2 DH domain (T264K, T264R, E266_M267delins, M267K, M267R, M267T and T376S; Figure 1C) that have thus defined the SOS2-specific NS9 subtype of this syndrome (OMIM #616559) [5,40]. In general, the clinical findings of NS patients harboring SOS2 mutations are similar to those with SOS1-mutated genes, although some SOS2-realted variants appear as rare cases of NS with particular predisposition for lymphatic complications [39]. The most benign lymphatic pathologies in SOS2-mutated patients involve lymphedema of lower limbs and genitalia, as well as congenital chylothorax. More severe complications that may even cause the death of some patients included chronic, progressive lymphedema with associated chylothorax, pleural effusions, and chronic lymphopenia [39]. A recent phenotype–genotype correlation study has revealed the association between mutant variations of SOS2 in NS patients with lower diastolic and systolic blood pressures, and lower percent of body fat [64]. The first prenatal case of NS with SOS2 mutations was reported in a euploid fetus with a severe increase in nuchal translucency and other relevant anomalies noticeable at ultrasound study, as well as markers of aneuploidies, caused by a de novo heterozygous missense mutation in SOS2 gene (c.800 T > A; p.M267K) [40].

### 3.2. SOS2 in Sporadic Cancers

Although mutated SOS2 has not yet been recognized as a cancer driver, at least 253 mutations in the SOS2 gene (195 missense, 45 synonymous, 12 truncating, and one splice-site) have been detected so far in sporadic tumors (https://www.intogen.org/search?gene=SOS2 (accessed on 20 June 2021)). In this regard, direct exome sequencing has detected the presence of missense-activating mutations in SOS2 in a small percentage of gallbladder carcinomas [65], and also in a subtype of desmoplastic melanomas [66]. Recent analysis of gene expression profiles has also reported MuD-dependent upregulation of SOS2 expression in cohorts of TCGA glioblastomas (GBM), and a correlation between high expression of the two genes and longer survival of proneural GBM patients [67]. Finally, a whole-exome sequencing analysis carried out on non-small cell lung cancer samples demonstrated a direct correlation between SOS2 and resistance mechanisms to Osimertinib [38].

These observations certainly warrant further evaluation of SOS2 as a potential therapeutic target for oncogenic processes in vivo. In this regard, single SOS2 depletion did not show any therapeutic benefit in a model of chemically-induced skin carcinogenesis but combined SOS1/2 depletion exhibited significantly stronger beneficial effects in comparison to single SOS1-KO mice [21], supporting at least a partial functional involvement of SOS2, and its consideration as a potential therapy target, in RAS-driven tumors. Consistent with this notion, genetically-mediated silencing of the human SOS2 gene by means of miRNAs or CRISPR/Cas9 also produces significant therapeutic benefits in different in vitro models, including human tumor cell lines (Table 2).

**Table 2:** **SOS2 gene silencing strategies in different tumor cell types**. List of genetically-based strategies to downregulate SOS2 gene in the indicated tumor cell lines. Resulting phenotypic consequences of SOS2 disruption are shown in the corresponding column.

SOS2 Disrution Strategy	Tumor Cell Line	Phenotypic Effect	Reference
*miR-148a*	BJAB and DG-75 and U2932 (B cell lymphoma and Burkitt lymphoma)	Reduction of ERK activation	[68]
*miR-148a-3p*	A549, HCC827 (lung cancer)	Reduction of proliferation and EM transition	[69]
*miR-193a-3p*	HEK293, SKOV3, and OVCA433 (ovarian cancer)	Suppression of MAPK–ERK signal transmission	[70]
*SOS2 KO mice*	MEFs expressing mutant RAS isoforms: HRAS^G12V^, NRAS^G12V^, or KRAS^G12V^	Impairment of RTK-dependent AKT phosphorylation Dispensable for RTK-dependent ERK activation	[31]
*CRISPR/Cas9*	H358 NSCLC cells (lung cancer)	Revert the transformed phenotype of KRAS mutant cells. SOS2 participates in anchorage-independent, but not in anchorage-dependent, growth.	[31,32]
*CRISPR/Cas9*	H23 NSCLC cells (lung cancer)	SOS2 participates in anchorage-independent growth. Reduce cell viability.	[32]
*CRISPR/Cas9*	SW620 (colorrectal cancer)	SOS2 participates in anchorage-independent growth.	[32]
*CRISPR/Cas9*	NCI-H1299 NSCLC cells (lung cancer)	SOS2 participates in anchorage-independent growth.	[32]
*CRISPR/Cas9*	YAPC cells (pancreatic cancer)	Revert the transformed phenotype of KRAS oncogenic cells.	[31]

Although oncogenic RAS proteins are constitutively activated (not needing, in theory, the action of upstream GEFs to become GTP-loaded), different reports have demonstrated that the cross-activation of wild-type RAS (which is SOS-dependent) by oncogenic mutated RAS is of critical importance for tumorigenic development in mutant RAS-driven tumors [71,72]. Regarding this, it is highly relevant to mention recent experimental evidence indicating that SOS1 and SOS2 may play specific, nonoverlapping functions in RAS-driven oncogenic cells. In particular, it has been reported that RTK–SOS2–WT RAS signaling, but not allosteric SOS2 activation, is a critical mediator of mutant KRAS-driven transformation [31] by protecting KRAS-mutated cancer cells from anoikis [32]. Consistent with the notion that SOS1 and SOS2 may promote distinctive control of differential aspects of wild-type RAS signaling in oncogenic RAS-driven tumors, the same group has also reported a hierarchical requirement for SOS2 to drive mutant RAS-dependent transformation, with KRAS being the most SOS2-dependent form (KRAS > NRAS > HRAS) [57].

### 3.3. SOS2 in Non-Tumoral Pathologies

Reports linking SOS2 alterations with other non-tumoral pathologies are very limited but specific. Thus, SOS2 has been proposed as a susceptibility locus for initiation of Alzheimer’s disease [73]. In particular, two single nucleotide polymorphisms (SNPs) were characterized in SOS2 that are significantly associated with late-onset Alzheimer’s disease, suggesting that SOS2 may be a male specific risk factor for Alzheimer’s disease [73]. Mutations in SOS2 have also been reported in association with metabolic cutis laxa disease [74]. GWAS analysis also supports genetic association between SOS2 and chronic periodontitis-related pathologies, especially in adults [75], as well as processes of elevated intraocular pressure (lead SNP rs72681869; G > C) [76].
